# Salt-inducible Kinase Regulation of Adipose Tissue Metabolism

**DOI:** 10.1210/endocr/bqaf092

**Published:** 2025-05-19

**Authors:** Fubiao Shi, Vineet Agrawal, Timothy A McKinsey, Sheila Collins

**Affiliations:** Division of Cardiovascular Medicine, Department of Medicine, Vanderbilt University Medical Center, Nashville, TN 37232, USA; Division of Cardiovascular Medicine, Department of Medicine, Vanderbilt University Medical Center, Nashville, TN 37232, USA; Tennessee Valley Healthcare System Nashville Veteran Affairs Hospital, Nashville, TN 37212, USA; Department of Medicine, Division of Cardiology and Consortium for Fibrosis Research & Translation, University of Colorado Anschutz Medical Campus, Aurora, CO 80045, USA; Division of Cardiovascular Medicine, Department of Medicine, Vanderbilt University Medical Center, Nashville, TN 37232, USA; Department of Molecular Physiology and Biophysics, Vanderbilt University, Nashville, TN 37212, USA

**Keywords:** salt-inducible kinase, adipocytes, glucose uptake, lipid metabolism, thermogenesis, cardiometabolic disease

## Abstract

Salt-inducible kinases (SIKs) are a subfamily of the adenosine monophosphate-activated protein kinase-related kinase family. To be activated, SIKs require phosphorylation in the catalytic kinase domain by liver kinase B1. In response to extracellular stimulations, their activity can be further regulated through phosphorylation by protein kinase A (PKA), and Ca^2+^/calmodulin-dependent protein kinases. PKA-mediated SIK inhibition is a major link between G-protein coupled receptor activation and the target gene transcription program. All 3 SIK isoforms—SIK1, SIK2, and SIK3—are expressed in adipocytes, with SIK2 being the most abundant in both rodents and humans. SIKs play essential roles in maintaining adipose tissue homeostasis by regulating physiological processes involving insulin signaling, glucose uptake, lipogenesis, and thermogenesis. Each SIK isoform could play both redundant and unique roles in these physiological processes. Many of the substrates that mediate their physiological functions in adipocytes have been characterized, and downstream mechanisms of action have also been proposed. However, due to the functional redundancy of SIKs, a major challenge is to delineate their isoform-specific roles in adipose tissue in vivo using genetic mouse models. In addition, common genetic variants and rare mutations in the SIK genes have been identified to be associated with metabolic, cardiovascular, and developmental conditions, suggesting a translational implication for human disease that deserves investigation. Furthermore, small molecular SIK inhibitors have been developed and have shown therapeutic potential in multiple disease areas. Evaluation of their metabolic and cardiovascular effects will be required for future clinical development of SIK inhibitors.

Salt-inducible kinases (SIKs) are serine/threonine kinases and belong to the adenosine monophosphate-activated protein kinase (AMPK)-related kinase family. The SIK subfamily consists of 3 members in vertebrates: SIK1, SIK2, and SIK3. SIK1 was first cloned from the rat adrenal gland after high-salt diet treatment using PCR-based cDNA subtraction hybridization (1). SIK2 and SIK3 were identified later based on their sequence similarity through in silico studies (2, 3). SIK genes are widely expressed in many tissues with isoform-specific enrichment patterns. SIK1 mRNA is highly expressed in the adrenal gland (1, 4). SIK2 mRNA is most abundant in the adipose tissue (2). SIK3 mRNA is more broadly expressed and is relatively enriched in the brain (5).

SIKs possess a conserved kinase domain in the N-terminal region, followed by a ubiquitin-associated domain and a regulatory C-terminal domain. SIK kinase activity is subjected to “yin-yang” regulation by a battery of upstream kinases, including liver kinase B1 (LKB1), protein kinase A (PKA), and Ca^2+^/calmodulin-dependent protein kinases (CaMKs) (Fig. 1). To be activated, SIKs require LKB1 phosphorylation of a conserved threonine residue in the activation loop of the kinase domain (Thr182 in SIK1, Thr175 in SIK2, and Thr221 in SIK3) (6). Phosphorylation of SIKs by PKA induces the binding of 14-3-3 proteins, which leads to allosteric regulation and inactivation of SIKs (7). Multiple PKA phosphorylation sites have been identified in SIK1 (Thr473, Ser575), SIK2 (Ser343, Ser358, Thr484, Ser587), and SIK3 (Thr469, Ser551, Ser626) (8). In response to extracellular hormonal stimulations that increase intracellular cAMP, SIKs become inactivated following phosphorylation by PKA. So, in essence, PKA phosphorylation of SIKs opposes their “activation” functions. This PKA-mediated SIK inhibition is a major link in a broader array of Gs-coupled G protein-coupled receptors and the target gene transcription program (9, 10). In addition, SIKs can be modulated by CaMKs through calcium-dependent signaling, although most of the literature on this role mainly describes it in other tissues instead of adipocytes. For example, phosphorylation of SIK1 by CaMK I at Thr322 increases SIK1 activity (11), and phosphorylation of SIK2 by CaMK I/IV at Thr484 leads to the degradation of SIK2 protein (12). Taken together, the regulation of SIKs by cAMP and calcium-dependent signaling cascades allows the dynamic tuning of SIK activity to coordinate physiological regulation in response to extracellular hormonal stimulation.

**Figure 1.**
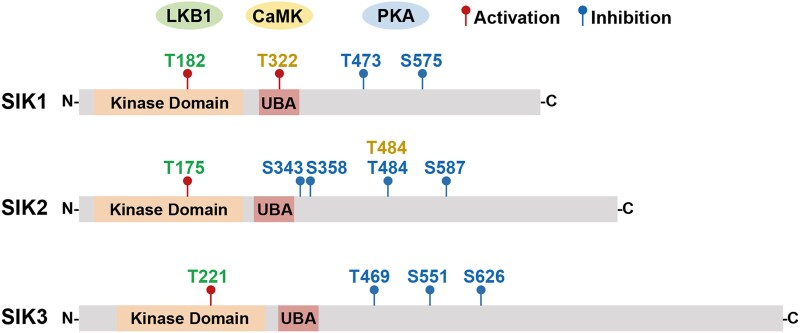
SIK protein structure and phosphorylation sites. The phosphorylation sites are labeled in green for LKB1, yellow for CaMKs, and blue for PKA. The effects of phosphorylation are denoted as red for activation and blue for inhibition. The amino acid residues refer to human proteins SIK1 (NP_775490.2, 783 aa), SIK2 (NP_056006.1, 926 aa), and SIK3 (NP_079440.3, 1321 aa), respectively. Abbreviations: CaMK, Ca^2+^/calmodulin dependent kinases; LKB1, liver kinase B1; PKA, protein kinase A; SIK, salt-inducible kinases; UBA, ubiquitin-associated domain.

SIKs phosphorylate a consensus motif of LXB(S/T)XS*XXXL (B = basic amino acid; X = any amino acid) in their substrates (13), which obviously is a relatively loose—perhaps even “promiscuous”—signature. The most well-characterized SIK substrates include CREB-regulated transcription coactivators (CRTCs) (13) and class IIa histone deacetylases (HDACs) (14, 15). SIK phosphorylation leads to the binding of 14-3-3 proteins and cytoplasmic retention of their substrates (13, 16). Extracellular stimulation that increases intracellular cAMP leads to the PKA-dependent phosphorylation and inactivation of SIKs. SIK inhibition consequently allows their substrates to be dephosphorylated and translocated to the nucleus to control target gene transcription programs. CRTCs interact with CREB to promote the transcription program involving a variety of metabolic processes such as gluconeogenesis (17), lipogenesis (18), and steroidogenesis (3). As traditionally understood, class IIa HDACs function together with transcription coregulators such as the myocyte enhancer factor 2 to control many developmental and physiological processes, including bone formation (19), myogenesis (20), and cardiac hypertrophy (21). Despite their weak intrinsic deacetylation activity toward histones (22, 23), class IIa HDACs can associate with HDAC3 and the nuclear receptor corepressor 2/silencing mediator for retinoid and thyroid-hormone receptors as an active complex to regulate gene transcription (24).

It has been increasingly appreciated that SIKs are important regulators of metabolic homeostasis [reviewed in (9, 10, 25, 26)]. They have been shown to involve the regulation of glucose and lipid metabolism in multiple tissues, such as the liver (17, 18, 27), pancreatic islets (28, 29), skeletal muscle (15, 30), and adipose tissue. In this review, we summarize the metabolic function of SIKs with a primary focus on the adipose tissue, including earlier work on their role in insulin signaling, glucose uptake, and lipogenesis and recent progress related to their role in adipocyte thermogenesis. In addition, we discuss recent literature related to the functional redundancy of SIKs in mouse models and emerging signaling mechanisms and substrate action in regulating adipocyte function. We further summarize the available human genetic evidence that implicates a role for SIKs in human disease and discuss the therapeutic potential of SIK inhibitors in treating obesity and cardiometabolic disease.

## Metabolic Function of SIKs in Adipose Tissue

### Insulin Signaling

SIK2 was first discovered through an in silico study based on its sequence similarity to SIK1. In mice, *Sik2* mRNA is highly expressed in the white adipose tissues and brown adipose tissues (BAT) (2). In 3T3-L1 adipocytes, *Sik2* mRNA levels increase significantly during differentiation (2). Its expression pattern is similar to that seen for the early response of transcription factors for adipogenesis, such as the CCAAT/enhancer binding protein β, indicating a potential role for SIK2 expression in adipogenesis (31).

**Figure 2.**
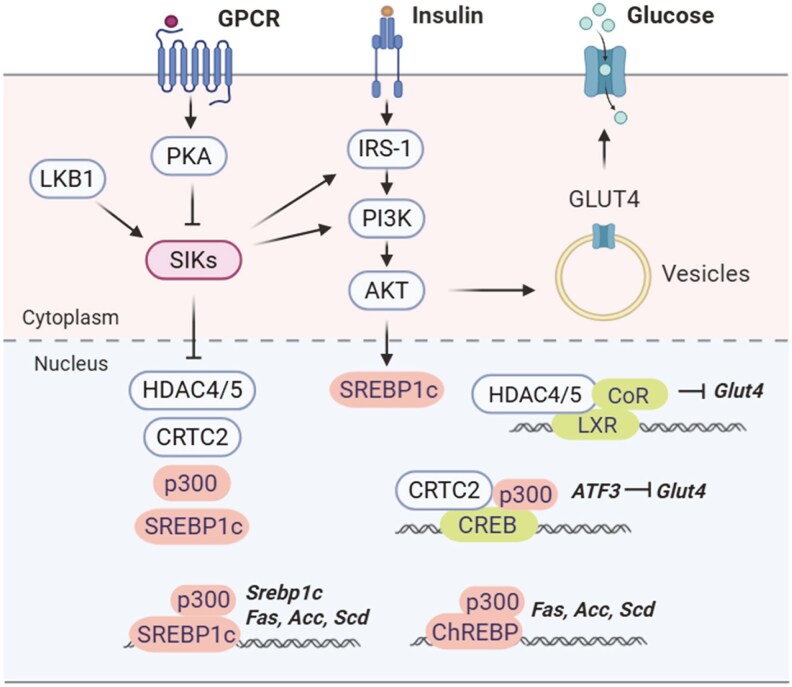
SIK regulation of adipocyte insulin signaling, glucose uptake, and lipogenesis. SIKs phosphorylate insulin receptor substrate 1 and p85α subunit of PI3K to control insulin signaling. SIKs promote *Glut4* gene expression by suppressing the nuclear translocation of HDAC4/5 and CRTC2. HDAC4/5 cooperate with transcription corepressor (CoR) to suppress *Glut4* expression, and their recruitment depends on the liver X receptor binding sites in *Glut4* promoter. CRTC2/CREB promotes the transcription of ATF3, which suppresses *Glut4* transcription. SIKs suppress the lipogenesis gene program by inhibitory phosphorylation on p300 and SREBP1c. Abbreviations: SIK, salt-inducible kinases; SREBP1c, sterol regulatory-element binding protein-1c.

Studies have shown that SIKs can regulate adipocyte insulin signaling and action through multiple layers of mechanisms (Fig. 2). SIK2 has been shown to phosphorylate insulin receptor substrate 1 (IRS-1) at Ser794 (human)/Ser789 (mouse) in vitro and in cultured 3T3-L1 adipocytes (2). From these findings, it was proposed that the phosphorylation of IRS-1 by SIK2 impacts the efficiency of insulin signaling cascades and renders the cells resistant to insulin stimulation in certain pathological conditions (31). However, subsequent studies did not support an inhibitory role for SIK2 in insulin signaling (32, 33). Instead, insulin-stimulated AKT phosphorylation is reduced in the adipose tissue of global *Sik2* knockout mice (32) and in human adipocytes treated with a pan-SIK inhibitor HG-9-91-01 (33). The preadipocytes from *Sik2* knockout mice show increased adipogenic potential but reduced insulin sensitivity (32). It has been noted that the IRS-1 Ser794 (human)/Ser789 (mouse) site can also be phosphorylated by AMPK (10). Pharmacological activation of AMPK by AICAR, which is admittedly not fully specific for AMPK (34), has been shown to increase insulin signaling by enhancing the association of IRS-1 with phosphoinositide 3-kinase (PI3K) (35). These studies suggested that SIK2 functions as a positive regulator of insulin signaling in adipocytes (10).

### Glucose Uptake

GLUT4 is the major glucose transporter for insulin-stimulated glucose uptake in adipocytes and skeletal muscle (36). In the absence of insulin, GLUT4 is sequestered in specialized immobile GLUT4 storage vesicles (36). Insulin stimulates exocytosis of GLUT4 from multiple intracellular compartments, which results in increased GLUT4 levels at the plasma membrane for glucose uptake into the cells (36).

Studies have shown that SIK2 can promote transcription of the *Glut4* gene by suppressing nuclear translocation of CRTC2 and HDAC4/5 in adipocytes (Fig. 2). On the other hand, the activation of CRTC2/CREB induces the transcription factor ATF3 and leads to the downregulation of *Glut4* gene transcription in adipocytes (37). HDAC4/5 also downregulates *Glut4* transcription in response to increased cAMP signaling in cultured adipocytes (38). It has been shown that the recruitment of HDAC4/5 requires the liver X receptor binding site in the *Glut4* promoter (38). In line with a positive role for SIK2 in *Glut4* gene expression, silencing of SIK2 decreases while silencing of CRTC2 or HDAC4 increases GLUT4 protein levels in 3T3-L1 adipocytes (39). Consistent with this, *Glut4* expression was reduced in adipocytes from *Sik2* knockout mice (32), and glucose uptake was reported to be reduced in primary rat adipocyte treated with a pan-SIK inhibitor (38).

It has been reported that the activity of SIK2 is increased in the adipose tissues of diabetic *db/db* mice (2) but downregulated in obese *ob/ob* mice (40). This disparity could be due to the different genetic background of *db/db* (C57BL/KsJ) and *ob/ob* (C57BL/6J) mice, which is an important distinction between these 2 genetic models that is not often appreciated [see (41) and references therein]. In humans, studies have shown that the expression and activity of SIK2 and SIK3 are downregulated in the adipose tissue of individuals with obesity (33). Specifically, SIK2 protein levels and its kinase activity in human adipocytes display a negative correlation to body mass index (33). Functional studies show that silencing of SIK2 in human adipocytes reduces insulin-stimulated glucose uptake and blunts Akt phosphorylation (33). A recent study in human adipocytes further showed that SIK2 is required for glucose uptake and insulin signaling (40). Pharmacological SIK inhibition leads to reduced glucose uptake in primary human adipocytes (40). Mechanistically, it has been proposed that SIK2 is required for insulin action, likely at the level of phosphatidylinositol (34,5)-trisphosphate generation or breakdown (40). In addition, the connection of SIK2 and the insulin signaling pathway has been reported in a separate study in ovarian cancer cells, which showed that SIK2 can phosphorylate the p85α regulatory subunit of PI3K at Ser154 to activate the PI3K/AKT signaling activity (42), again supporting SIK2 as a positive regulator of insulin signaling.

### Lipid Metabolism

It has been shown that SIK1 and SIK2 can negatively regulate hepatic lipogenesis through different mechanisms (Fig. 2). SIK1 suppresses hepatic lipogenesis by inhibitory phosphorylation at Ser329 of sterol regulatory-element binding protein-1c (SREBP-1c) (27), a master transcriptional regulator of the lipogenesis gene program (43). Overexpression of SIK1 suppresses while knockdown of SIK1 increases hepatic expression of lipogenic genes, such as fatty acid synthase (*Fas*) and acetyl-CoA carboxylase (*Acc*) (27). Coexpression of phosphorylation-resistant SREBP-1c with SIK1 restored hepatic lipogenic gene expression and triacylglycerol levels (27). In contrast, SIK2 represses lipogenic gene expression by inhibitory phosphorylation of histone acetyltransferase p300 at Ser89 (18), which prevents the carbohydrate-response element binding protein from activating the hepatic lipogenic gene program (18).

In adipocytes, studies have shown that SIK2 controls the lipogenic gene program by regulating SREBP-1c (44). For example, knockdown of *Sik2* in 3T3-L1 adipocytes induces the expression of lipogenic genes such as *Fas*, *Acc2*, and stearoyl-CoA desaturase (*Scd*) (44). Overexpression of SREBP-1c can rescue the SIK2-dependent inhibition of *Fas* expression (44). In line with this, the expression of lipogenic genes such as *Fas* and *Acc1* is increased in the adipose tissue of *Sik2* knockout mice (32). Of note, genetic mouse models of *Sik2* have shown various adiposity phenotypes. Global *Sik2* knockout mice have comparable body weight to the wild-type controls when fed either a chow diet or a high-fat diet (HFD) (32). However, transgenic mice that express a constitutively active SIK2 S587A mutation in BAT are prone to diet-induced obesity (45). The mechanisms by which this sustained SIK2 activity leads to diet-induced obesity might involve a coordinated alteration in insulin action, glucose uptake, and lipogenesis in the adipose tissue. However, the transgene expression in the latter study (45) was driven by the fatty acid-binding protein 4 (*Fabp4*) promoter, which is not very specific to adipocytes (46). Thus, these results need to be interpreted with caution.

It has been reported that SIK2 is also involved in fatty acid oxidation (FAO) (32, 42). The expression of FAO genes is reduced in the liver and skeletal muscle of global *Sik2* knockout mice, such as acyl-CoA oxidase 1 (*Acox1*), carnitine palmitoyltransferase-1 (*Cpt-1*), and medium-chain acyl-CoA dehydrogenase (*Mcad*) (32). Another study reported that SIK2 positively regulates the FAO gene program in ovarian cancer metastasis (42). SIK2 is highly expressed in the adipocyte-rich metastatic deposits of ovarian cancer (42). Transgenic overexpression of SIK2 in ovarian cancer cells promotes abdominal metastasis, while SIK2 depletion prevents metastasis in vivo (42). Mechanistically, it has been proposed that adipocytes induce calcium-dependent activation and autophosphorylation of SIK2, and the activated SIK2 plays a dual role in promoting AMPK-induced phosphorylation of ACC and in activating the PI3K/AKT pathway (42). SIK2 inhibition significantly reduced the adipocyte-mediated increase in *CPT-1* expression, indicating that SIK2 was required for fatty acid oxidation in omental metastases (42). This study suggested that SIK2 is the key regulator of the adipocyte-induced signaling cascades in metastatic cancer cells (42).

### Thermogenesis

The adipocyte thermogenic gene program is orchestrated by a coordinated signaling cascade induced by extracellular neuronal and hormonal stimulations that drive the expression of a panel of genes governing adipocyte thermogenic and mitochondrial biogenesis. For instance, as 1 of the most potent thermogenic stimulators, cold temperature induces the release of norepinephrine from the sympathetic nerve system, which act on the β-adrenergic receptors (βARs) in the adipocyte and stimulate the cAMP-mediated signaling cascade to dive the expression of uncouple protein 1 (*Ucp1*) and peroxisome proliferator activated receptor gamma coactivator 1 alpha (*Pgc1α*), which are key regulators of adipocyte thermogenesis and mitochondrial biogenesis (47).

**Figure 3.**
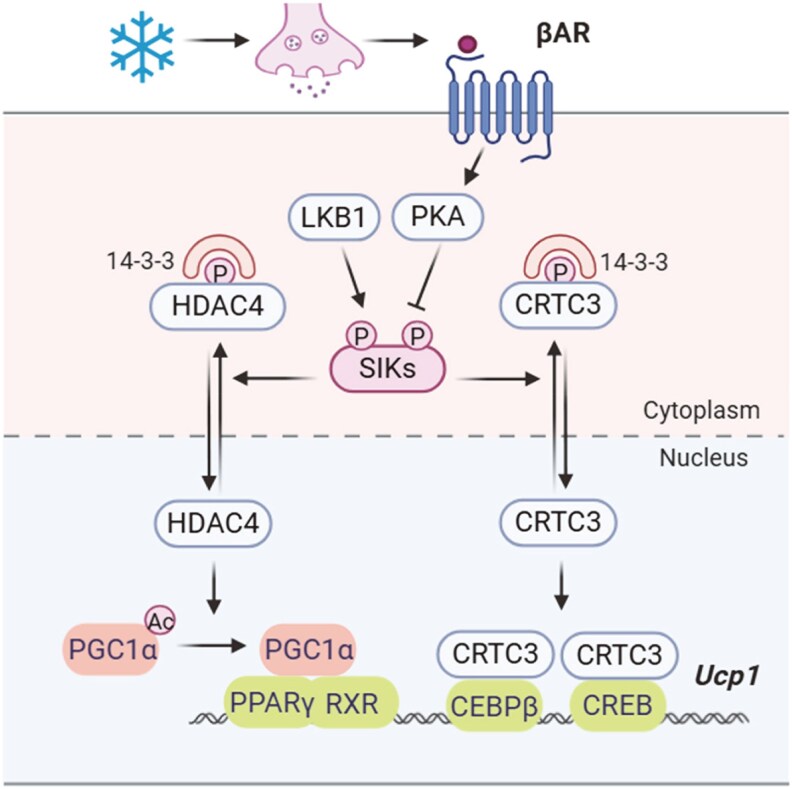
SIK regulation of adipocyte thermogenic gene expression. LKB1 phosphorylates and activates SIKs. SIKs phosphorylate HDAC4 and CTRTC3, inducing their 14-3-3 protein binding and cytoplasmic retention. Cold temperature stimulates sympathetic nerve system release of norepinephrine and acts through β-adrenergic receptor signaling to inhibit SIK activity via PKA. SIK inhibition leads to dephosphorylation and nuclear translocation of HDAC4 and CRTC3. HDAC4 interacts and deacetylates PGC1α, which promotes PGC1α co-activator activity to drive *Ucp1* gene expression. CRTC3 promotes the recruitment of C/EBPβ and cooperates with CREB to stimulate *Ucp1* gene expression. Abbreviations: C/EBPβ, CCAAT/enhancer binding protein β; SIK, salt-inducible kinases.

SIKs play essential roles in mediating the βAR signaling cascade to control the adipocyte thermogenic gene program (Fig. 3). At baseline without cold stress, SIKs are constitutively active upon phosphorylation by LKB1. Active SIKs phosphorylate their substrates such as HDACs and CRTCs, which leads to their cytoplasmic retention. βAR stimulation induces the PKA-mediated phosphorylation of SIKs in the regulatory domain and leads to SIK inactivation. SIK substrates such as HDACs and CRTCs hereby become dephosphorylated and translocate to the nucleus, where they promote the transcription program of thermogenic genes, along with the other transcriptional regulators that respond to the cAMP/PKA activity.

An early study by Muraoka and colleagues showed that the expression of *Ucp1* and *Pgc1a* is increased in a brown adipocyte cell line T37i after insulin treatment (45). This induction of gene expression could be inhibited by overexpression of a constitutively active SIK2 S587A mutant or a dominant-negative CREB (45). This study suggests the involvement of SIK2 in the regulation of *Ucp1* and *Pgc1a* expression in brown adipocytes. However, the mechanism for this transcriptional regulation of thermogenic gene expression is unclear and might be unique to this T37i cell line.

Studies with genetic mouse models implicated a role for SIKs in the adipocyte thermogenic gene program. For example, genetic deletion of the *Lkb1* gene in adipocytes blunts SIK activity (since LKB1 is an important kinase activating SIKs) and leads to activation of the brown adipocyte gene program (48, 49). Adipocyte-specific *Lkb1* knockout mice have increased BAT mass, enhanced subcutaneous inguinal white adipose tissues (iWAT) browning, and higher energy expenditure (48). They also have improved glucose tolerance and insulin sensitivity and are more resistant to HFD-induced obesity (48). Mechanistically, it has been proposed that *Lkb1* deletion leads to the nuclear translocation of CRTC3 to recruit the transcription factor CCAAT/enhancer binding protein β for *Ucp1* transcription (48). In addition, it has also been proposed that *Lkb1* gene deletion also suppresses AMPK activity, which leads to activation of the mammalian target of rapamycin (mTOR) signaling pathway and subsequent BAT expansion (48). It was shown in another study that HDAC4 is also involved in the induction of beige adipocytes in the absence of *Lkb1* (49). Treatment with the β_3_AR agonist CL-316,243 induces dephosphorylation of HDAC4 at Ser245 in the iWAT (49). HDAC4 is also hypophosphorylated in *Lkb1* knockout adipocytes, mimicking the effects of β-adrenergic stimulation in the iWAT (49). Combined deletion of *Hdac4* and *Lkb1* in adipocytes reverts the beige (brown-like) fat expansion and thermogenic gene expression (49). Interestingly, in the BAT, LKB1 controls thermogenic capacity independently of HDAC4 (49), suggesting depot-specific mechanisms for thermogenic activation upon *Lkb1* gene deletion. In addition, there are no phenotypic differences observed in *Hdac4* adipocyte knockout mice compared with control mice, suggesting that HDAC4 is not essential for the basal activity of adipose tissue thermogenesis (49).

We recently reported that pharmacological SIK inhibition promotes the adipocyte thermogenic gene program and adipose tissue browning (50). In agreement with a previous study (51), we showed that treatment with pan-SIK inhibitors HG-9-91-01 and YKL-05-099 increased thermogenic gene expression in primary adipocytes and brown adipocyte cell lines (50). In addition, treatment of mice with YKL-05-099 promoted the thermogenic gene expression and adipose tissue browning (50). We further showed that HDAC4 is a major mediator of the SIK inhibitor's effects to drive thermogenic gene expression. Knockdown of *Hdac4* diminished the induced *Ucp1* gene expression after SIK inhibitor treatment (50). In addition, HDAC4 interacts with and deacetylates PGC1α (50), which potentially increases its coactivator activity to promote thermogenic gene expression (52).

As summarized earlier, the metabolic function of SIKs in the adipocyte has been well documented in both rodents and humans by studies of various models. In the following sections, we discuss several important topics related to functional redundancy of SIKs, new signaling mechanisms and substrate actions, and translation of research evidence from laboratory animals to humans. While the primarily focus is on the adipocytes, literature from other tissues is included as additional support.

## Functional Redundancy of SIK Isoforms in Mouse Models

The 3 SIK isoforms are widely expressed in many tissues (5). SIK isoforms appear to have overlapping roles in regulating substrate action and metabolic processes. This could be due to their conserved kinase activity toward shared substrates and similar phosphorylation mechanisms that modulate SIK kinase activity. However, each SIK isoform could also have a distinct function that might not be compensated for by the other members. This could relate to the variations in their regulatory domain and selectivity toward regulatory kinases and downstream substrates. This also depends on the isoform-specific variations in their gene expression, protein abundance, and subcellular location in each tissue or cell type. For example, nuclear localization sequences have been reported in SIK1 but not in SIK2 or SIK3 (53). SIK1 expression is more dynamically regulated through the CRTCs-CREB transcription regulators in response to neuronal and hormonal stimulation (54). In contrast, SIK2 and SIK3 are more constitutively expressed, but their expression can still be modified under certain disease and diet conditions (33, 55).

It has been shown that SIKs play redundant roles in regulating adipocyte function and metabolism. SIK2 is highly expressed in both rodent and human adipocytes compared with SIK1 and SIK3. Adipocyte-specific *Sik1* knockout mice appear normal on both chow and HFD (30). Global *Sik2* knockout mice are mildly glucose and insulin intolerant and show several functional alterations in the adipose tissue, including increases in lipolysis, lipogenic gene expression, adipocyte size, and macrophage infiltration and decreases in *adiponectin* and *Glut4* expression (32). Global *Sik3* deletion leads to skeletal defects, and more than half of the knockout mice die prematurely (55). The surviving knockout mice show metabolic dysfunction characterized by a malnourished phenotype (ie, lipodystrophy, hypolipidemia, hypoglycemia, and hyperinsulin sensitivity) accompanied by cholestasis and cholelithiasis (55). However, it is unclear if adipose tissue is primarily responsible for such metabolic defects.

It has also been shown that SIKs have redundant roles in regulating adipose tissue browning. The expression of the key thermogenic gene *Ucp1* did not change in the iWAT from either *Sik1* or *Sik2* global knockout mice but was elevated in the iWAT of *Sik1* and *Sik2* double knockout mice even without additional β-adrenergic stimulation (49). At thermoneutrality, *Sik2* knockout mice exhibited enhanced thermogenic response in a *Ucp1*-dependent manner (56). In HIB-1B brown adipocytes, knockdown of either *Sik1*, *Sik2*, or *Sik3* increases the thermogenic gene expression without additional β-adrenergic stimulation (50). These data suggest that SIKs play redundant roles as downstream components of βAR signaling to control adipocyte thermogenic gene expression. Characterization of the mouse models with individual or compound *Sik* gene deletions in adipocytes will help elucidate the function of SIKs in adipose tissue in vivo.

The redundant functions of SIKs in vivo have also been documented in other tissues. For example, in the liver, deletion of *Sik1* or *Sik2* alone in mice does not impact hepatic gluconeogenesis (17, 30), suggesting that SIKs play a redundant role in hepatic gluconeogenesis. In macrophages, pharmacological SIK inhibition leads to their polarization toward an anti-inflammatory phenotype (57). Characterization of primary macrophages derived from mice with single or double loss of function mutations in *Sik* genes established that all 3 SIK isoforms contribute to macrophage polarization, with a major role for SIK2 and SIK3 (57). In addition, SIKs mediate the intracellular actions of PTH 1 receptor signaling to control bone development and remodeling (58). SIK3 plays a major role in chondrocyte differentiation and hypertrophy, which is further augmented by SIK1 and SIK2 (58). Combined deletion of *Sik2* and *Sik3* in osteoblasts and osteocytes led to a dramatic increase in bone mass that phenocopies a constitutively active PTH 1 receptor mutation that causes Jansen's metaphyseal chondrodysplasia (58). This was not observed in mice with individual osteoblast and osteocyte-specific deletion of *Sik1*, *Sik2*, or *Sik3* or in mice with compound deletion of *Sik1/2* and *Sik1/3* (58), suggesting that SIK2 and SIK3 play a major role in maintaining bone homeostasis.

The distinct functions of SIK isoforms have also been demonstrated by their roles in insulin secretion and fibrogenesis. SIKs regulate insulin secretion in pancreatic islets through isoform-specific mechanisms (28, 29). SIK1 *inhibits* insulin secretion by phosphorylation of phosphodiesterase 4D, which reduces cellular cAMP concentrations (28). In contrast, SIK2 *promotes* insulin secretion by phosphorylation of cyclin-dependent kinase 5 regulatory subunit 1, which leads to the suppression of cyclin-dependent kinase 5 activity and activation of voltage-dependent Ca^2+^ channels (29). In addition, SIK2 plays a unique role in fibrogenesis (59). The tyrosine kinase inhibitor nintedanib has been approved for the treatment of idiopathic pulmonary fibrosis, but it can also inhibit SIK kinase activity (59). A study showed that genetic deletion of SIK2, instead of SIK1 or SIK3, protects against bleomycin-induced fibrosis (59). Treatment with a selective SIK2 inhibitor ARN-3236 prevented fibroblast activation and extracellular matrix gene expression in human fetal lung fibroblasts and attenuated bleomycin-induced pulmonary fibrosis in mice (60). Whether SIKs might also have isoform-specific roles in regulating metabolic processes in adipocytes will need to be characterized by using appropriate genetic mouse models in future studies.

## Novel Signaling Mechanisms and Substrate Action in Metabolic Regulation

PKA-mediated SIK inhibition is a key link between G protein-coupled receptor activation and target gene transcription programs. By regulating the cytoplasmic nuclear shuttling of SIK substrates, this signaling module has been implicated in diverse physiological processes. Recent studies have identified a novel connection between SIK3 and the mTOR signaling network. In 2018, Csukasi and colleagues reported a new skeletal dysplasia caused by a homozygous mutation in the catalytic domain of SIK3 (Arg187Cys) and observed decreased activity of mTORC1 and mTORC2 due to accumulation of DEPTOR, a negative regulator of both mTOR complexes (61). This study showed that SIK3 is a positive regulator of mTOR signaling, which functions by triggering DEPTOR degradation in response to PTH/PTH-related protein during skeletogenesis (61). In a separate study from our group, we sought to characterize the phosphoproteomic profile of brown adipocytes after insulin and β-adrenergic stimulation to screen for βAR signaling-specific mTORC1 substrates in brown adipocytes (50, 62). Our study identified a specific phosphorylation site in SIK3 (Ser884), whose phosphorylation is increased by βAR stimulation and blocked by rapamycin but not affected by insulin (50), suggesting a possible mTORC1-dependent phosphorylation event. Our data further showed that SIK3 interacts with Raptor, the defining component of mTORC1, suggesting that SIK3 is physically associated with mTORC1 (50). At present, the functional consequence of SIK3-mTORC1 interaction in adipocytes is still under investigation.

As AMPK-related kinases, SIKs require phosphorylation by LKB1 to be active. However, they have very distinct physiological functions compared to AMPK [reviewed by Sakamoto et al (10)]. Their activity is regulated in response to extracellular hormonal signals but not by nucleotides like an energy sensor (10). While AMPK regulates multiple metabolic enzymes to maintain cellular adenosine triphosphate levels, SIKs primarily regulate gene expression by controlling the cytoplasmic-nuclear shuttling of their substrates (10). Unlike SIKs, AMPKs have very distinct functions in adipocytes. Inducible deletion of the AMPK β-subunits in adipocytes leads to cold intolerance and impaired brown and beige fat function and exacerbates diet-induced hepatic steatosis and insulin resistance (63). Thus, adipocyte AMPK is vital for maintaining mitochondrial integrity and thermogenic capacity and protects against diet-induced obesity (63). Deletion of *Lkb1* in adipocytes leads to the inactivation of AMPK-related kinases, including AMPK and SIKs. However, adipocyte *Lkb1* knockout mice show increased brown fat mass and beige fat formation (48, 49), suggesting that SIK inactivation has a dominant role upon *Lkb1* deletion in adipocytes. It was proposed that the increased brown fat mass could be due to the increased mTORC1 activity induced by AMPK inactivation (48). Since AMPK and mTOR are master metabolic regulators, understanding the connection of SIKs with these signaling networks would provide novel insights.

New mechanisms of SIK substrate actions have also been reported. HDAC7 has been identified as a SIK1 substrate that promotes pathologic cardiac remodeling (64). This role is distinct from the stress-induced CaMK/protein kinase D-dependent phosphorylation for HDAC4/5/9 in pathological cardiac remodeling (65). In a recent study from our group, we identified PGC1α as a new HDAC4 substrate to mediate the SIK's inhibitory effect to induce the adipocyte thermogenic gene expression (50). A similar connection has also been implicated in an earlier study for the HDAC4-PGC1α axis in maintaining skeletal muscle homeostasis (66). In addition to class II HDACs and CRTCs, SIK substrates identified in other tissues or cells, such as protein phosphatase methylesterase 1 (11), histone acetyltransferase p300 (18), phosphodiesterase 4D (28), cyclin-dependent kinase 5 regulatory subunit 1 (29), the p85α subunit of PI3K (42), and nuclear receptor corepressor 2/silencing mediator for retinoid and thyroid-hormone receptor (67), might also be involved in SIK regulation of adipocyte function. Furthermore, unbiased methods will be necessary in future studies for the discovery of novel SIK substrates to fully understand its mechanisms of action in the adipocytes and other tissues.

## Translation From Laboratory Animals to Humans: Implication for Human Disease

Rare genetic mutations and common single nucleotide polymorphisms (SNPs) in the SIK genes have been reported to be associated with human disease (summarized in Table 1). A missense SNP rs3746951 encodes a Gly15 to Ser (G15S) mutation in human SIK1 (68). It has been shown that the G15S mutation increases SIK1 activity and is associated with higher plasma membrane Na^+^/K^+^-ATPase activity, lower blood pressure, and a decrease in left ventricular mass (68). A homozygous mutation in the catalytic domain of SIK3 (Arg187Cys, rs1565460853) impairs SIK3 activity and leads to skeletal dysplasia by altering mTOR activity downstream of the PTH/PTH-related protein signaling during skeletogenesis (61).

**Table 1.** Disease-associated genetic variants in SIK genes

Gene	SNP	Allele	Mutation	Protein function	Trait	References
SIK1	rs3746951	C > T	Gly15Ser	Gain of function	Lower blood pressure	(68)
SIK3	rs1565460853	C > T	Arg187Cys	Loss of function	Skeletal dysplasia	(61)
SIK3	rs10047459	C > T	Intronic	N/A	Higher triglycerides	(69)
SIK3	rs533556	A > C	Intronic	N/A	Higher triglycerides	(69)
SIK3	rs139961185	G > A	Intronic	N/A	Higher postprandial triglycerides	(70)
SIK3	rs12225230	G > C	Pro1136Arg	Unknown	Higher ApoA1 and HDL	(71-73)

Abbreviations: SIK, salt-inducible kinases.

SNPs in the human SIK3 gene have been reported to be associated with dyslipidemia in genome-wide associated studies. Braun et al reported that 2 intronic SNPs, rs10047459 and rs533556 in SIK3, are located within a linkage disequilibrium block in chromosomal region 11q23.3 and show strong signals associated with higher triglycerides in Asian Indians (69). Ko et al used a cross-population allele screen approach and identified that an intronic SNP rs139961185 in SIK3 is an Amerindian-specific genetic risk factor for dyslipidemia or obesity and underwent positive selection in Mexican populations (70). It was further shown that the SIK3 risk variant carriers display high triglyceride levels after a high-fat meal (70), suggesting a potential functional relevance. In addition, a missense SNP rs12225230 in SIK3 was identified to be associated with increased plasma concentrations of high-density lipoprotein cholesterol and apolipoprotein A1 in several genome-wide associated studies (71-73). Although the association of SNPs in SIK3 with dyslipidemia is well documented, the functional relevance of these variants in lipid metabolism is yet to be established.

The difference in SIK gene expression between rodents and humans needs to be considered to ascertain if the studies of rodent models can appropriately inform human physiology and disease. In adipocytes, the 3 SIK isoforms show comparable expression between rodents and humans, with SIK2 the most abundant and SIK1 and SIK3 relatively lower (33, 49). However, discrepancies in gene expression in certain disease conditions have been reported between different mouse strains and humans (2, 33, 40). These disparities indicate that SIK gene expression might be subject to unique regulation in different genetic backgrounds or disease conditions. As already noted, SIK activity is regulated by posttranslational modification through protein phosphorylation; alterations in gene expression should be interpreted with caution to better inform the functional consequences.

It has been shown that active BAT is associated with better cardiometabolic outcomes in both humans and mice (74, 75). Therefore, targeting the thermogenic fat to promote energy expenditure has been considered a promising strategy for the treatment of obesity (76). However, targeting the thermogenic pathway by selective β_3_AR agonist in adipocytes has been shown to lead to adverse side effects in humans (77-79). As discussed in this review, an increasing body of literature has shown the metabolic effects of SIK inhibitors in adipocytes, including their effects on insulin signaling (33), glucose uptake (40), and adipocyte thermogenesis (50, 51). Although it is yet to be determined if SIK inhibitors will have similar effects on adipocyte thermogenesis in humans, the literature suggests SIK inhibitors could be a potential new reagent to target these metabolic pathways in adipocytes. Again, further investigations are required to determine whether the evidence from cell and mouse models can be translated into human studies in the future.

## Therapeutic Potential of SIK Inhibitors for Cardiometabolic Disease

Small molecule SIK inhibitors have been developed and shown therapeutic potential in multiple disease areas, such as inflammation (80-84), fibrosis (60), osteoporosis (19, 85, 86), acute myeloid leukemia (87), and certain types of cancer (42, 88, 89) (summarized in Table 2). Several SIK inhibitors have been advanced to clinical trials for inflammatory disease (81-83) and breast and ovarian cancer (88). Although SIKs are important regulators of fuel metabolism [reviewed in (9, 10, 25, 26)] and cardiovascular function [reviewed in (88, 90)], the metabolic and cardiovascular effects of SIK inhibitors have been less documented in preclinical mouse models and clinical studies. Understanding the cardiometabolic effects of SIK inhibitors will lead to a new avenue for the treatment of obesity and related cardiometabolic diseases. In addition, probing the isoform-specific functions of SIKs using genetic mouse models will provide new biology to inform the development of selective SIK inhibitors in the future.

**Table 2.** Treatment effects of SIK inhibitors in disease models

Compounds	Selectivity	Disease	Treatment effects	References
ARN-3236	SIK2	Fibrosis	Inhibits bleomycin-induced lung fibrosis	(60)
ARN-3236	SIK2	Cancer	Inhibits ovarian cancer growth	(42)
ARN-3261	SIK2	Cancer	Inhibits breast and ovarian cancer	(88)
GLPG3312	SIK1, SIK2, SIK3	Inflammation	Anti-inflammatory and immunomodulation	(82)
GLPG3970	SIK2, SIK3	Inflammation	Immunomodulation in a colitis model	(81)
GLPG4399	SIK3	Inflammation	Immunomodulation in arthritis models	(83)
JRD-SIK1/2i-4	SIK1, SIK2	Inflammation	Ameliorates disease in a colitis model	(84)
OMX-0407	SIK3	Cancer	Inhibits breast cancer in mouse models	(89)
SK-124	SIK2, SIK3	Osteoporosis	Increases bone formation	(86)
YKL-05-099	SIK1, SIK2, SIK3	Inflammation	Anti-inflammatory and immunomodulation	(80)
YKL-05-099	SIK1, SIK2, SIK3	Osteoporosis	Increases bone formation	(19, 85)
YKL-05-099	SIK1, SIK2, SIK3	Leukemia	Inhibits AML progression	(87)
YKL-05-099	SIK1, SIK2, SIK3	Metabolism	Increases adipose tissue thermogenesis	(50)

Abbreviations: AML, acute myeloid leukemia; SIK, salt-inducible kinases; SNP, single nucleotide polymorphism.

## Data Availability

Data sharing is not applicable to this article as no datasets were generated or analyzed.
